# Biomolecular Transformations Shape the Environmental Fate of Nanoscale and Emerging Materials

**DOI:** 10.1021/acs.accounts.5c00587

**Published:** 2025-10-22

**Authors:** Swaroop Chakraborty, Iseult Lynch

**Affiliations:** † School of Geography, Earth & Environmental Sciences, 1724 University of Birmingham, Edgbaston B15 2TT, United Kingdom; ‡ Centre for Environmental Research and Justice, 1724 University of Birmingham, Edgbaston B15 2TT, United Kingdom

## Abstract

Engineered nanomaterials (ENMs) have revolutionized biomedicine, energy, and environmental remediation due to their unique physicochemical properties. However, these properties are not static; they evolve dynamically as ENMs interact with real-world biological and environmental systems. Central to this transformation is the formation of the biomolecular corona, a dynamic layer of adsorbed proteins, lipids, and metabolites that govern how nanomaterials interface with their surroundings. The corona alters the surface chemistry, colloidal stability, and biological identity of an ENM, ultimately dictating its environmental fate, functionality, and safety profile, but also evolves as the surroundings change or as the living system responds to the presence of the nanomaterials and secreted biomolecules.

Over the past decade, our research has elucidated how biomolecule-driven transformations, such as dissolution, ion release, sulfidation, enzymatic degradation, and redox reactions, can be modulated by the acquired corona. These processes not only determine the longevity and toxicity of nanomaterials but also offer programmable opportunities for safe degradation or detoxification. For instance, coronas can enhance or suppress ion leaching and catalyze phase changes into less bioavailable forms.

We have also explored the role of eco-coronas, formed in environmental matrices like soil or aquatic systems, which contain a broader range of biomolecules beyond proteins, such as humic acids, polysaccharides, and microbial secretions. These coronas initiate transformation cascades as ENMs transition through different environmental compartments, influencing mobility, speciation, and bioavailability to organisms. Through this lens, we view ENMs not as inert entities but as evolving systems shaped by dynamic biological interactions.

While the biomolecular corona concept is well-established for engineered nanomaterials such as metal and polymeric nanoparticles, it is now extending to emerging materials such as metal–organic frameworks (MOFs). These hybrid, porous materials are increasingly used in biomedical, catalytic, and environmental applications, yet their transformations under biological and ecological conditions remain largely uncharted. We argue that applying corona concepts to MOFs provides a powerful lens to anticipate their environmental fate and guide safe-and-sustainable design. Our recent work demonstrates that protein coronas can either stabilize or destabilize MOFs, modulate enzyme function, or even program degradation via enzyme-sensitive linkers. These findings provide the foundation for safe-by-design and corona-informed design strategies, where materials are engineered to respond predictably to biological cues.

This Account integrates advances in *in situ* characterization, machine learning, and predictive modeling to chart a path toward programmable, safe, and sustainable (by design) ENMs. By embracing corona dynamics as a tool, not just a challenge, materials that perform their intended function and then degrade into benign byproducts at the end of their lifecycle can be designed. We anticipate that leveraging biomolecule-driven transformations will become a cornerstone of safe nanomaterial design, aligning innovation in nanotechnology with principles of environmental and human health protection.

## Graphical abstract

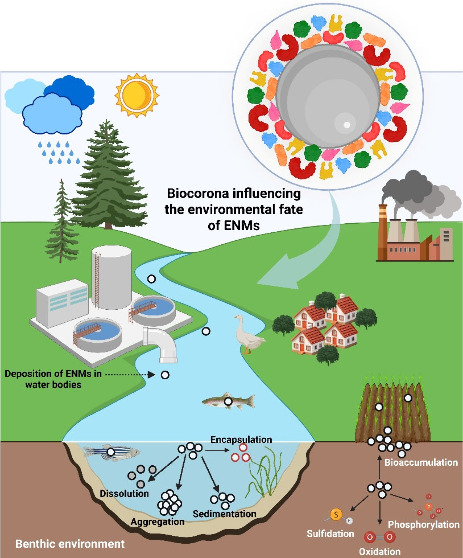

## Key References

-

Cedervall, T. ; Lynch, I. ; Lindman, S. ; Berggard, T. ; Thulin, E. ; Nilsson, H. ; Dawson, K. A. ; Linse, S. Understanding the Nanoparticle–Protein Corona Using Methods to Quantify Exchange Rates and Affinities of Proteins for Nanoparticles. Proc. Natl. Acad. Sci. U.S.A. 2007, 104 (7), 2050–2055.17267609 10.1073/pnas.0608582104PMC1892985 ^1^ First multimethod quantification of nanoparticle–protein corona dynamics that established competitive affinity-based binding, exchange rates and stoichiometries, revealing weakly bound versus strongly bound protein layers and biofluid-dependent “biological identity”. This methodological foundation underpins current mechanistic nanotoxicology across media and matrices.
-

Wheeler, K. E. ; Chetwynd, A. J. ; Fahy, K. M. ; Hong, B. S. ; Tochihuitl, J. A. ; Foster, L. A. ; Lynch, I. Environmental Dimensions of the Protein Corona. Nat. Nanotechnol 2021, 16 (6), 617–629.34117462 10.1038/s41565-021-00924-1 ^2^ First-of-its-kind synthesis extending protein corona science to environmental systems, defining eco-coronas, contrasting clinical and ecological contexts, mapping implications for nanomaterial transformation, uptake, trophic transfer, and ecotoxicity, and charting methodological priorities to predict, control, and steer coronas for safer, sustainable nanotechnology globally.
-

Chetwynd, A. J. ; Lynch, I. The Rise of the Nanomaterial Metabolite Corona, and Emergence of the Complete Corona. Environ. Sci. Nano 2020, 7 (4), 1041–1060.^3^ Establishes the metabolite corona and proposes the “complete corona”, arguing that metabolites coexist with proteins, shaping signaling and toxicity, necessitating integrated proteomics–metabolomics workflows. Reframes exposure/fate prediction and safe-by-design strategies beyond the protein-centric paradigm, with early evidence and a research roadmap.
-

Ellis, L. J. A. ; Lynch, I. Mechanistic Insights into Toxicity Pathways Induced by Nanomaterials in *Daphnia magna* from Analysis of the Composition of the Acquired Protein Corona. Environ. Sci. Nano 2020, 7 (11), 3343–3359.^4^ First in-depth eco-corona study in model organism *Daphnia magna* linking corona composition on pristine versus aged nanomaterials to mechanistic toxicity pathways; aging reduced bound proteins and cytotoxic signatures, indicating adaptive homeostasis shifts, providing pathway-level readouts for ecotoxicity assessments across media/materials.
-

Chakraborty, S. ; Menon, D. ; Mikulska, I. ; Pfrang, C. ; Fairen-Jimenez, D. ; Misra, S. K. ; Lynch, I. Make Metal–Organic Frameworks Safe and Sustainable by Design for Industrial Translation. Nature Reviews Materials 2025, 10 (3), 167–169.^5^ Presents a framework for emerging materials like MOFs, integrating chemical, physical, biomolecular, and biological transformations into a hierarchical lifecycle perspective; operationalizes SSbD to design recyclable MOFs, translating nanocorona insights toward industrial deployment under realistic environmental stresses, with thresholds and levers.

## 1 Introduction

Engineered nanomaterials (ENMs) have emerged as a transformative tool across a range of fields, including biomedicine, energy storage, and environmental remediation.^6^ Their unique physicochemical properties, such as high surface area-to-volume ratio, tunable reactivity, and multifunctionality, underpin their versatility. Despite their promise, ENMs face challenges of stability, safety, and environmental impact. A primary challenge is the dynamic nature of ENMs, which undergo transformations upon interaction with real-world environments. These transformations, which include dissolution, agglomeration, ion exchange, enzymatic degradation, and structural modifications, alter the materials physicochemical properties,^7^ influencing their functionality, biocompatibility, and potential toxicity. For instance, metallic NM dissolution releases toxic ions, while enzymatic degradation can yield harmful byproducts.^8^ Understanding and predicting these transformation pathways is vital for developing ENMs that are high-performing, safe and sustainable.

Central to these transformation processes is the formation of the acquired biomolecular corona, a dynamic layer of proteins, lipids, and small biomolecules that absorb onto the surface of ENMs upon their introduction into biological or environmental matrices.^2,3^ The corona provides a biological or environmental surface layer, largely dictated by the underlying chemical nature of the ENM, that can mask its pristine properties. In most cases, this layer is composed of physiosorbed components that are reversibly bound, thereby dynamically modulating surface chemistry, reactivity, and biological interactions. For example, the corona can mediate ENM interactions with cellular membranes, regulate dissolution rates, or influence stability in dispersion. These processes determine how ENMs behave in biological and environmental systems, how they distribute in tissues, and their potential for long-term toxicity or environmental accumulation.

Biomolecule-driven transformations are particularly relevant to the safe and sustainable-by-design framework,^9^ which aims to integrate material safety into the development process. The growing recognition of the importance of biomolecular transformations is reflected in the rapid expansion of literature. Bibliometric mapping (Figure 1) highlights how the field has evolved from early studies on protein coronas to broader concepts such as biomolecular and eco-coronas, linking nanomaterials with biological identity, nanomedicine, and environmental systems. This intellectual trajectory underscores that transformations mediated by coronas are not peripheral phenomena but central to determining nanomaterial behavior and fate. By leveraging the properties of the biomolecular corona, researchers can control and predict the behavior of ENMs in complex environments, to minimize unintended consequences and design ENMs that undergo controlled, benign transformations at the end of their functional life. Such transformations align with sustainability principles, addressing growing concerns over ENM accumulation in ecosystems and the associated risks to human and environmental health.

**Figure 1:**
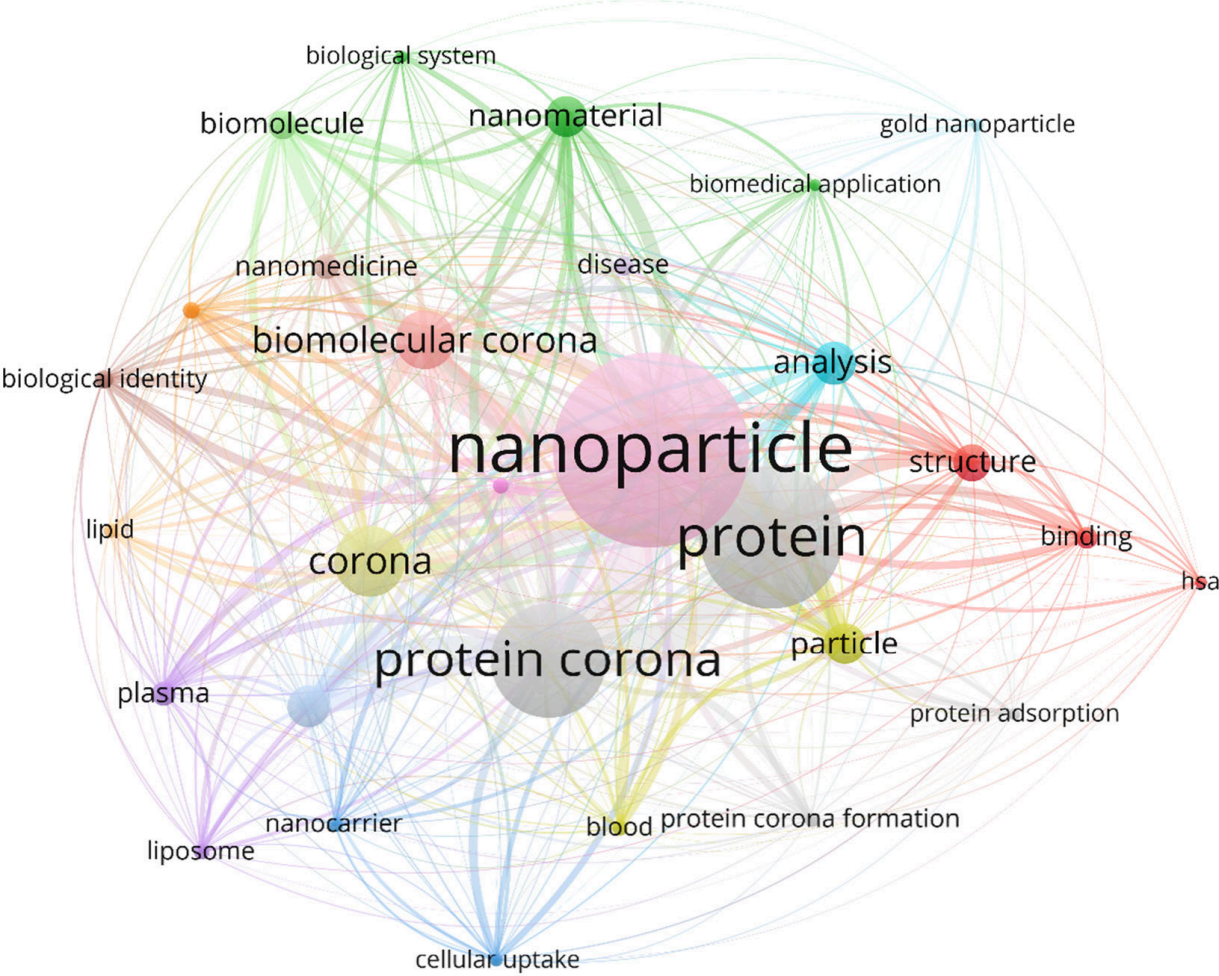
Bibliometric co-occurrence network of keywords related to the biomolecular corona, generated using VOSviewer (version 1.6.20; van Eck and Waltman).^10^ The data set was constructed from publications indexed in *Web of Science* (Clarivate Analytics) from 2007 to 2024 using the query terms “protein corona”, “biomolecular corona”, and “nanoparticle corona”. Node size reflects frequency of occurrence. Link thickness represents co-occurrence strength, and colors indicate distinct thematic clusters. The visualization highlights the central role of *nanoparticle*, *protein*, and *protein corona* in the field while also capturing the emergence of broader concepts such as *biomolecular corona*, *biological identity*, *lipids*, *nanomedicine*, and *eco-coronas*. This bibliometric mapping illustrates the intellectual structure and evolution of corona research, showing the shift from early protein-focused studies toward a systems-level understanding of nanomaterial transformations.

This Account focuses on biomolecule-driven transformations of ENMs, including dissolution, ion exchange, enzymatic degradation, and structural modifications. It highlights the pivotal role of biomolecular coronas in driving or modulating these processes, offering insights into how this knowledge can be applied to the design of safe and sustainable materials. The discussion extends to emerging materials like metal–organic frameworks (MOFs), exploring how lessons from traditional ENMs can inform their development and safe deployment.

We examine the evidence for how biomolecular coronas drive ENM transformations across biological and environmental systems and discuss how this knowledge can inform the rational design of safe and sustainable NMs that balance high performance with safety and environmental responsibility.

## 2 From Corona Formation to Transformation Pathways in Engineered Nanomaterials

### 2.1 Dynamic Protein Coronas: Interaction Lifetimes and Evolution

We begin by exploring the dynamic formation of the protein corona and its evolution that establishes a nanomaterial’s biological identity and influences subsequent transformation processes. One of the first aspects we explore is how the protein corona forms and evolves, laying the groundwork for subsequent transformations. ENMs are rarely bare in real-world environments or biological systems. The moment a nanomaterial (NM) enters a complex medium (whether blood plasma, cell culture, soil pore water, etc.), a *biomolecular corona* forms, a sheath of proteins, lipids, metabolites, and other molecules absorbed to its surface.^11,12^ Pioneering work by Cedervall, Lynch and colleagues revealed that this corona is not a static coating but a highly dynamic interface.^1,13^ Using techniques like fluorescence correlation spectroscopy, Cedervall et al. identified two kinetically distinct layers on poly-N-isopropylacrylamide-based NPs: a rapidly exchanging “soft” corona (forming within seconds to minutes) and a more tightly bound “hard” corona that develops over hours.^14^ Low-affinity proteins bind transiently, while high-affinity ones form longer-lived coronas. The progressive expansion of the corona concept, first introduced in 2007 from protein-centric views to multicomponent, eco- and metabolite coronas has shaped our current understanding and is illustrated in Figure 2.

**Figure 2:**
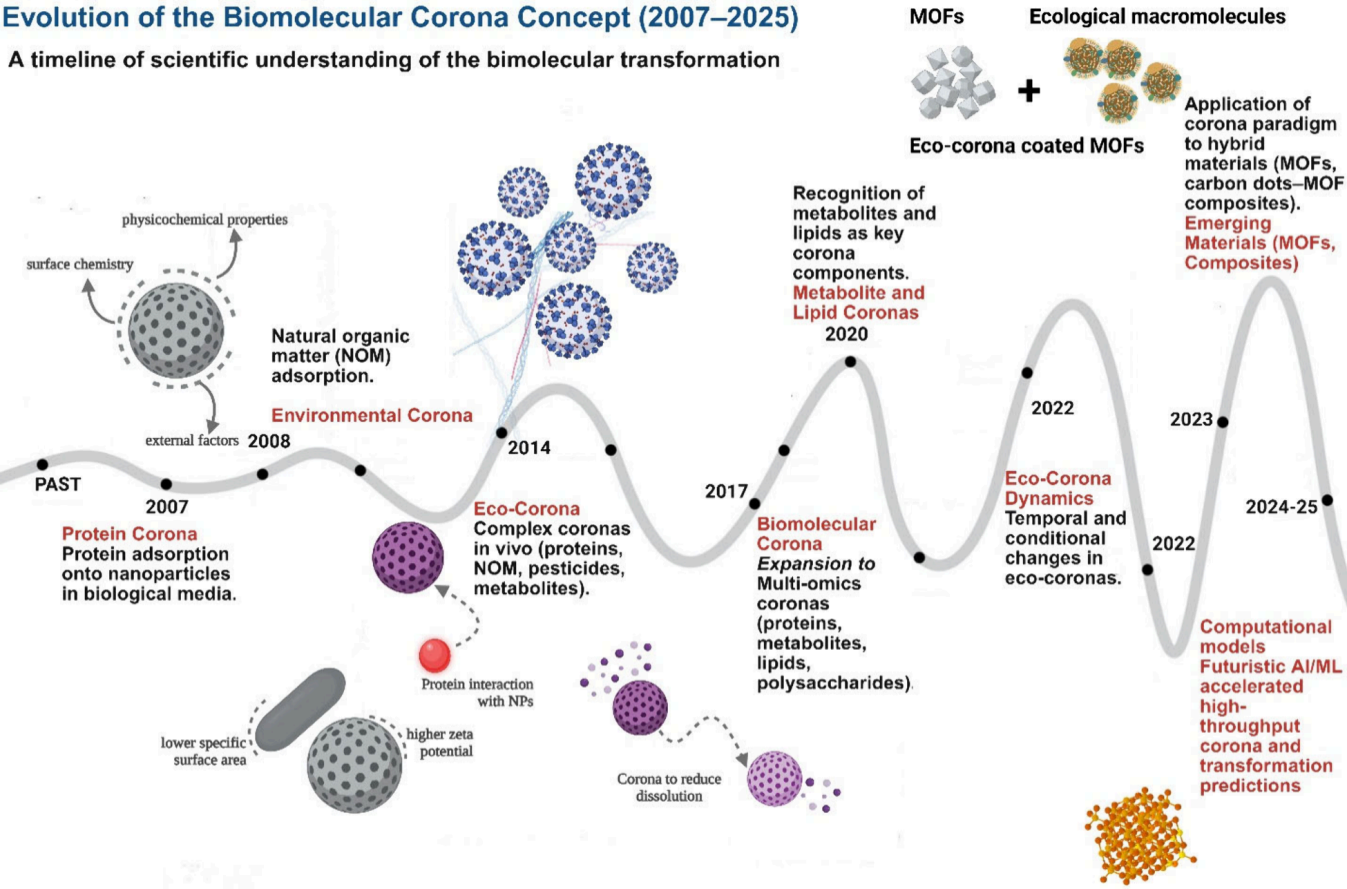
Evolution of the biomolecular corona concept (2007–2025). Early work focused on protein coronas (2007) and environmental coronas formed by natural organic matter (2008). The paradigm expanded to eco-coronas (2014) and biomolecular coronas (2017), incorporating proteins, lipids, metabolites, and polysaccharides. Recent advances highlight metabolite and lipid coronas (2020), dynamic eco-corona processes (2022), and cascade-driven transformations (2023). Emerging research extends the corona framework to hybrid and porous materials such as MOFs and composites, while future directions (2025−) point toward predictive, systems-level models integrating artificial intelligence/machine learning (AI/ML) and high-throughput multiomics analytics. Together, these milestones illustrate a shift from descriptive characterization to predictive understanding of how coronas mediate NM transformations across biological and environmental systems. Figure created using Biorender Software.

Importantly, the composition of the corona is selective and depends on NM’s properties. Our research demonstrated that NM *size* and *surface chemistry* strongly determine which proteins adsorb from plasma using six polystyrene NM variants (50 vs 100 nm, with plain, carboxylated, or amine-modified surfaces) each of which acquired a distinct hard corona fingerprint.^13^ Certain plasma proteins showed preference for specific sizes or surface charges, and the corona profiles differed markedly between the NMs with functional consequences. For instance, variations in the corona have been linked to differences in cellular uptake and immune recognition of NMs. A biological “identity” is essentially imprinted onto the NM by its corona, influencing how cells “see” and process the NM. As a striking example, a recent *in vitro* blood–brain barrier model showed that as gold nanoparticles passed from the “blood” side to the “brain” side, their corona composition underwent dramatic changes, and once beyond the barrier, the evolved corona determined the particles’ fate in the brain compartment.^11^ The initial corona formed in blood was *not* predictive of the NM’s interactions after crossing the barrier. As NMs progress through endosomes into the cytosol, some originally adsorbed proteins are replaced by cytosolic ones. For example, Cai et al. observed that enzymes like pyruvate kinase bound to gold NMs in the cytoplasm, displacing portions of the “blood” corona. This perturbed proteostasis and activated chaperone-mediated autophagy in cells.^15^ Such findings illustrate that corona dynamics are an ongoing process *in vivo*, with real consequences for cell health. Our research established that coronas are dynamic and selective, evolving over time and space to mediate NMs interactions with living systems. Having established how the corona forms and evolves, we next explore how it modulates critical physicochemical NMs transformations such as dissolution, redox reactions, and phase changes.

### 2.2 Modulation of Dissolution and Redox Transformations by Coronas

The corona defines biological identity and governs the chemical reactivity of ENMs by influencing ion release, redox behavior, and surface transformations. One of the most profound impacts of the biomolecular corona is on the physical and chemical transformation of NMs, notably dissolution, oxidation state changes, and precipitation reactions. Ellis and Lynch^4^ demonstrated that environmental aging of NMs substantially alters their acquired protein coronas, with measurable consequences for dissolution, redox transformations, and whole organism toxicity. Across Ag and TiO_2_ systems, freshly dispersed NMs exhibited the highest number of surface-bound proteins (up to 246 for pristine PVP-coated Ag NMs in artificial water containing natural organic matter (NOM)), many associated with ATP/GTP/DNA binding, mitochondrial breakdown, and oxidative stress pathways, such as copper–zinc superoxide dismutase, chorion peroxidase, and histone proteins. Such coronas indicate reactive surfaces that promote redox cycling and ion release. In contrast, 6-month aged (in medium) NMs consistently bound fewer proteins (e.g., ≤81 for aged PVP–Ag in HH combo medium), with enrichment in calcium-binding (e.g., calmodulin) and redox-homeostasis proteins (e.g., peroxiredoxins, apolipoprotein D), suggesting passivation of reactive sites via surface oxide formation, sulfidation, or NOM adsorption. Bioaccumulation data aligned with this pattern: freshly dispersed NPM showed up to 4-fold higher internalized metal concentrations in *Daphnia magna* than aged analogues, supporting reduced bioavailability and slower dissolution for aged forms.^4^ Collectively, these results link corona composition directly to the thermodynamics and kinetics of NM transformation, with protein signatures serving as functional biomarkers for the degree of oxidative reactivity and dissolution potential *in vivo*.

Similarly, for metallic NMs like silver (AgNPs), it is well-known that release of metal ions (Ag^+^) through dissolution is a primary driver of toxicity and reactivity.^16^ However, protein coronas can significantly mediate the dissolution behavior of AgNPs, in some cases, acting as a diffusional barrier that *slows* the release of ions, in others, accelerating dissolution by binding and removing released ions, shifting equilibrium. For example, a monolayer of bovine serum albumin (BSA) on AgNPs altered dissolution kinetics in a size-dependent manner: for 10 nm AgNPs, increasing BSA concentration enhanced the constant dissolution rate up to ∼7.7-fold (by sequestering Ag^+^ and preventing redeposition).^16^ Conversely, BSA coronas can protect AgNP surfaces from certain aggressive agents (like acidic or oxidative species), thereby dampening instantaneous dissolution under those conditions.^17^ These seemingly paradoxical effects highlight that corona–NM interactions control how, when, and where ions release: the corona can retard dissolution by shielding the surface or promote dissolution by binding/removing ions.

Corona formation also influences what new phases form on or around NMs. A landmark study by Miclăuş et al. showed that corona proteins drive the sulfidation (transformation of Ag^0^ or Ag^+^ to silver sulfide (Ag_2_S), an insoluble mineral) of AgNPs in biological media.^18^ In serum-containing medium, Ag^+^ ions released from AgNPs were trapped within the protein corona and converted into nanocrystalline Ag_2_S on the NP’s surface. Remarkably, the corona acted as a nanoreactor, concentrating Ag^+^ and supplying sulfur (from thiol groups in proteins like cysteine residues), thus facilitating the growth of Ag_2_S crystallites at the interface.^18^ The loosely bound corona proteins played an opposite role: they bound Ag^+^ and diffuse away, *preventing* it from being sulfidized near the particle. This highlights that the corona is not merely a passive “shield” but functions as an active chemical interface capable of catalyzing NM transformations. Context is crucial, however, as in a different environment, the corona might heighten NP reactivity. For instance, under the acidic, oxidative conditions of lysosomal fluids (which mimic intracellular digestion), even protein-coated AgNP can undergo rapid dissolution and oxidation.^16^ In low pH and high-peroxide media, AgNPs quickly release Ag^+^ despite the presence of a corona, leading to bursts of ions that can damage cells.^19,20^ Here the corona may be unable to fully protect the NM; instead, it might facilitate reactions by concentrating acidic or oxidizing molecules near the surface. The takeaway is that corona effects are context-dependent: a corona can either stabilize a NP or activate it, depending on the surrounding chemistry.

### 2.3 Biotransformation Cascades in Complex Systems

We now turn to how ENMs are transformed in complex systems, such as soil–plant–water environments, where corona composition and dynamics change along the exposure pathway. As investigations shift from simplified laboratory media to complex living systems and natural environments, a clearer understanding of multistage transformation cascades governed by dynamic and evolving coronas.^21^ These coronas, which form on the surface of NMs upon exposure to biological or environmental matrices, undergo continuous changes depending on the surrounding conditions. In particular, the concept of the “eco-corona” has gained prominence, referring to the layer of biomolecules derived from natural environments such as soil, water, or organismal secretions.^21^ The eco-corona plays a critical role in shaping the environmental fate, behavior, and potential toxicity of NMs, acting as a mediator between the material and its biological or ecological context.^2^ The eco-corona can include a plethora of biomolecules: polysaccharides, humic and fulvic acids, lipids, amino acids and proteins, metabolites, and even pollutants.^2,22^ Nasser and Lynch^21^ showed that natural proteins released by *D. magna* and its gut microbiome form an eco-corona on polystyrene NPs, which increased the particles’ uptake (as the particles agglomerated and resembled the algal food) and toxicity.

While proteins often dominate and impart a “biological identity” (e.g., signaling to receptors), other corona components are crucial for understanding environmental transformations.^22^ Our recent review thus argues for a “*complete corona*” perspective that integrates proteins with small molecule (sometimes called metabolites). Metabolites and natural organic matter can bind to NMs either directly or indirectly (via protein scaffolds) and can drastically influence transformations by altering surface chemistry or participating in reactions.^22^ For example, coronas rich in organic acids might promote leaching of metal ions, whereas lipid coronas could inhibit oxidation. Recognizing these contributions opens new “transformation pathways” beyond simple protein interactions. The vision of mapping the complete bio–eco-corona is driving new research tools (combining proteomics with metabolomics) to predict how NMs evolve in real-world scenarios.

Microbial communities further amplify these transformations. Biofilms—communities of microorganisms encapsulated in secreted extracellular polymeric substances (EPS)—are especially important. As reviewed by Mokhtari-Farsani, Lynch, and co-workers, the EPS matrix of biofilms can wrap around carbon-based NMs (like graphene oxide sheets or carbon nanotubes), forming a biocorona that disperses the NMs and also actively biodegrades them.^24^ Many bacteria and fungi produce enzymes (e.g., peroxidases, laccases) and generate Reactive oxygen species (ROS) as defense or metabolic byproducts. When a carbon nanotube gets lodged in a biofilm, enzymes such as horseradish peroxidase (HRP) in the vicinity oxidize the carbon lattice.^25^ The corona might include these enzymes or may simply position the nanotube in the EPS where ROS accumulates, *cleaving* the graphene sheets and carbon nanotubes, breaking them into smaller fragments.^26,27^ Over time, this can lead to complete mineralization of carbon NMs, converting them to CO_2_ and H_2_0,^28^ as demonstrated for HRP degradation of carboxylated single-walled nanotubes *in vitro* into oxidized polyaromatic fragments with CO_2_ gas release, evidencing total breakdown.^28^

Organism-derived coronas can directly drive multistep NM transformations in soil and water. For example, extracellular enzymes secreted by soil microbes (e.g., cellulase) rapidly coated TiO_2_ NPs with highly anionic coronas, causing strong electrostatic repulsion and drastically reducing NP deposition in soil matrices.^29^ Likewise, marine invertebrates (e.g., mussels) develop high salt (≈500 mM NaCl), protein-rich eco-coronas that induce TiO_2_ NPs agglomeration, profoundly altering NP transport in ocean food webs.^30^ Mechanistically, such corona constituents can catalyze chemical transformations; adsorbed proteins unfold or chelate surface metals, “exposing reactive residues” that drive NP dissolution or reprecipitation. Each corona-mediated transformation (dissolution, sulfidation, complexation, etc.) feeds the next, in a continuous cascade of transformations. Thus, coronas act as “reaction media”: they promote NP dissolution, aggregation or sulfidation and new phase formation, effectively linking successive stages of NM weathering in the environment.^2^

Such biotransformation challenges the notion that carbon NMs are biologically inert or persistent; instead, they behave as dynamic entities that microbes can *digest*, especially when guided by coronas that recruit the right biochemical tools. This synergy between corona formation and biodegradation is a frontier, shifting the narrative from NMs being simply pollutants to potentially biodegradable (or transformable) materials under the right environmental conditions.^3,4,31^ Even in scenarios without complete degradation, coronas influence intermediate transformations. In agricultural settings, for instance, ZnO NPs might dissolve in the mildly acidic root apoplast releasing Zn^2+^, which then gets sequestered by organic acids or phosphate to form Zn-organic complexes or Zn-phosphate precipitates within the corona milieu. The corona’s constituents (like organic acids or phospholipids) act as chemical reagents determining the speciation of Zn. Such speciation changes were shown for cerium oxide (CeO_2_) NPs in plant tissues whereby a phosphate-rich biomolecule corona resulted in partial transformation into cerium phosphate.^23^ Zhang et al. describe the lifecycle of a NP in the soil–plant system as a “transformation cascade” (Figure 3). The NP that first entered the soil is not the same chemical entity that reaches a leaf or is exuded by the plant roots; it has been continuously modified by corona interactions along the way.

**Figure 3:**
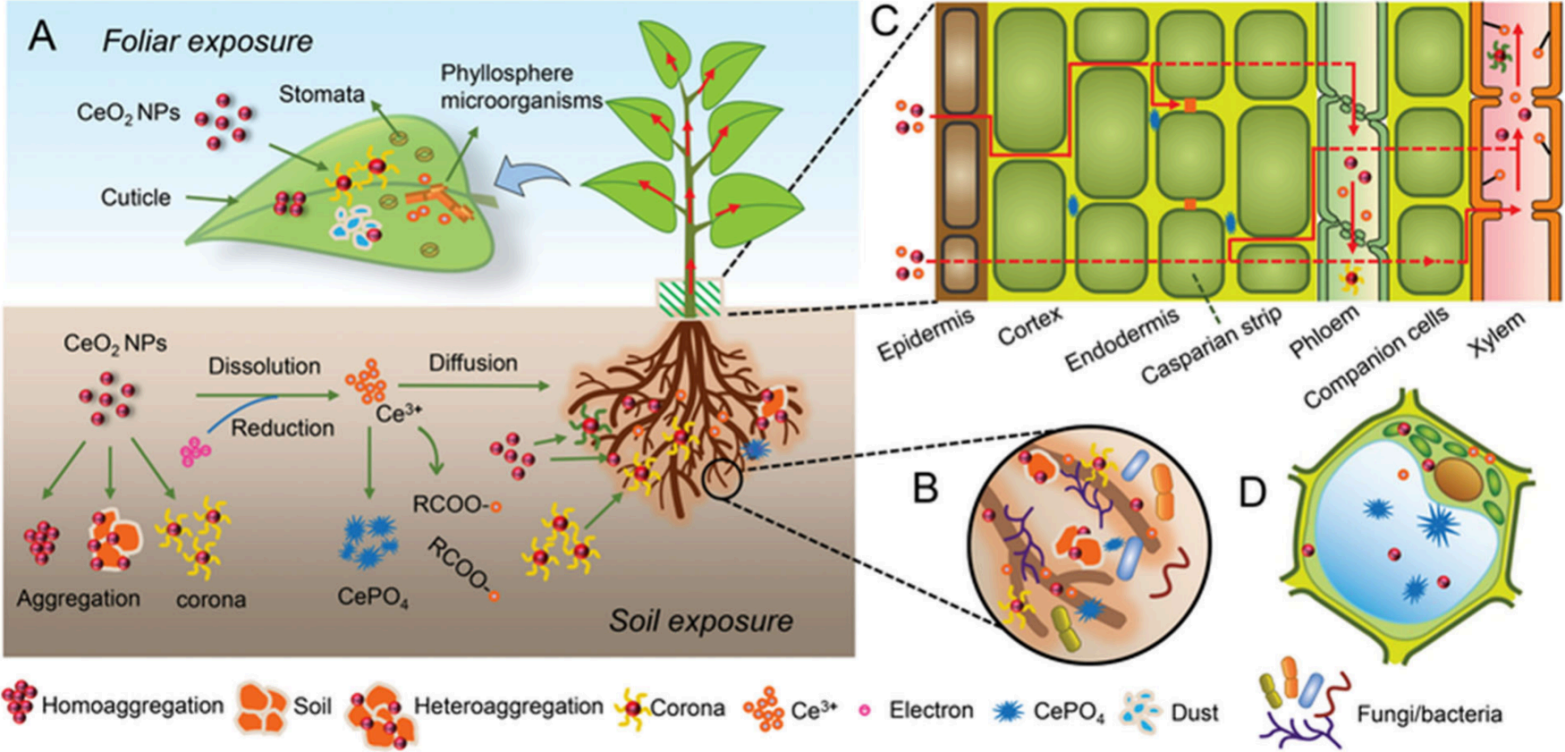
Schematic representation of NM transformation cascades in plant systems using CeO_2_ NPs as an example. (A) Pathways of foliar and soil exposure, including aggregation, corona formation, dissolution, reduction, and complexation to form CePO_4_. (B) Interaction of CeO_2_ NPs and their transformation products with soil microbes and extracellular polymers. (C) Uptake and translocation of NMs and ions across roots and shoot tissues, highlighting barriers such as the Casparian strip and transport through xylem and phloem. (D) Intracellular interactions of transformed Ce species with organelles, fungi, and bacteria. Together, these processes illustrate how coronas and biotic interfaces orchestrate multistep transformations and govern NP fate in complex biological-environmental systems. Figure reproduced from ref 23 with permission from Wiley-VCH, Copyright 2020.

These insights highlight the need for integrated analytical approaches to map entire biotransformation cascades. Multiomics corona profiling (proteomics + metabolomics) in series with spatially resolved spectroscopy (e.g., synchrotron X-ray speciation, nanoSIMS) can reveal the evolving composition and chemical state of NMs *in situ*. Isotope-labeling of NP components, coupled with *in situ* microscopy or X-ray imaging, can track dissolution and reprecipitation products at high resolution. Correlating corona fingerprints with geochemical models enables prediction of fate. By combining these emerging tools under a unified cascade framework, researchers can systematically chart how NMs are transformed stepwise as they pass through soils, organisms, and food webs.^2^

## 3 Experimental and Computational Approaches to Study Transformations

To unravel biomolecule-driven transformations of ENMs, our team and wider research group employs a suite of complementary experimental and computational techniques. Advanced analytical methods characterize the dynamic biomolecular corona and its influence on ENM behavior, while imaging techniques directly visualize transformations *in situ*. These efforts are coupled with predictive modeling tools that leverage data to forecast transformation pathways, providing a robust framework for characterizing and predicting ENM transformations in complex environments.

### 3.1 Biomolecular Corona Characterization

Proteomic analyses are central to understanding corona composition. Our early demonstration that the protein corona is highly selective, with each NP acquiring a distinct “fingerprint” of proteins depending on its size and surface chemistry.^13^ Today, high-resolution proteomics (e.g., LC-MS/MS or CE-MS/MS) is routinely used to identify and quantify dozens to hundreds of corona proteins. Our research has extended this to integration of metabolomics and lipidomics to capture the “complete corona” of proteins *and* small molecules that coat NPs.^35^ This holistic view has revealed that metabolite coronas can be as influential as protein coronas in modulating NM behavior. A wide suite of complementary techniques is now employed for corona analysis, spanning biochemical assays, imaging, and high-resolution spectroscopy (Figure 4). Together, these tools enable both *in situ* and *ex situ* insights into how coronas evolve and direct nanomaterial transformations.

**Figure 4:**
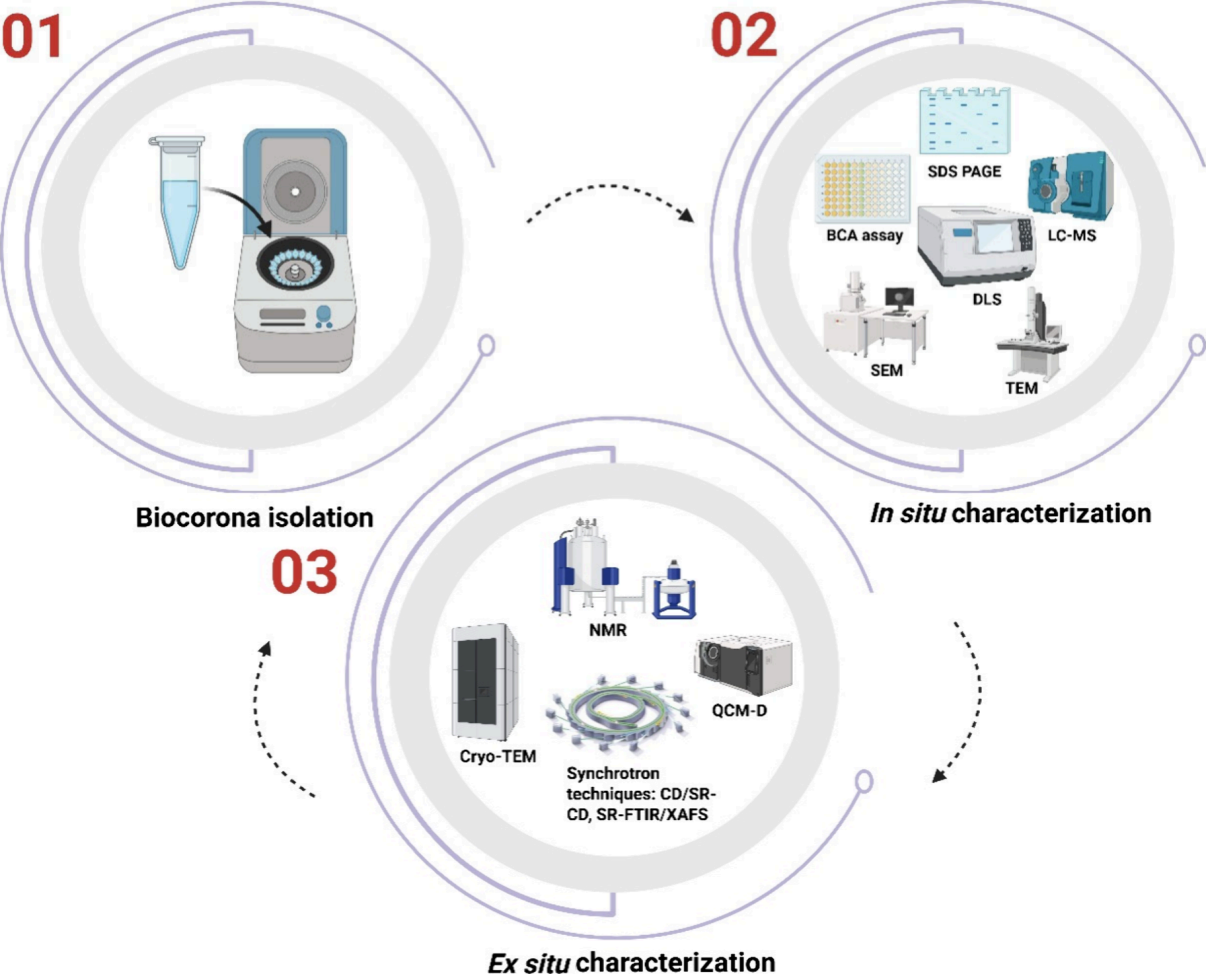
Workflow for biomolecular corona analysis. The process begins with biomolecular corona isolation (01), typically involving separation of nanoparticle–corona complexes from free biomolecules. This is followed by *in situ* characterization (02) using biochemical, spectroscopic, and microscopic tools (e.g., SDS-PAGE, BCA assay, LC-MS, DLS, SEM, TEM) to identify corona components and assess nanoparticle stability. *Ex situ* characterization (03) then applies advanced structural and biophysical methods, including Cryo-TEM, NMR, QCM-D, and synchrotron-based spectroscopies (CD, SR-CD, SR-FTIR, XAFS), to reveal structural evolution, binding mechanisms, and transformation pathways. Together, these complementary approaches provide a comprehensive picture of corona composition and its role in nanomaterial transformations. Figure created using Biorender Software.

For instance, Chetwynd et al. used quantitative metabolomics to demonstrate that NMs acquire a metabolite corona in biological fluids, with small molecules binding selectively to different NM surfaces and influencing subsequent cellular interactions.^32^ Such findings extend the concept of the corona from being protein-centric to a multilayered, dynamic interface that reflects the biochemical complexity of the surrounding environment. This comprehensive profiling maps what biomolecules are present and provides corona fingerprints that correlate with biological outcomes such as uptake pathways, biodistribution, or catalytic activity. By building reference databases of corona compositions under varying conditions, the community is moving toward predictive “corona fingerprinting” tools to classify NMs and anticipate their interactions. Importantly, our group has contributed to this standardization, developing reproducible digestion, identification, and quantification workflows that enable cross-study comparison of corona data sets, as well as reporting guidelines. Our group developed MINBE guidelines,^33^ emphasizing controlled exposure, corona isolation, high-resolution detection, and transparent analysis, enabling reproducible data sets for ML integration. For transformation studies, this framework prevents artifacts (e.g., false dissolution rates from protein loss during isolation) and enables cross-study validation.

Visualizing the biomolecular corona and its evolution on NMs demands a multimodal toolkit. As reported in our recent nature protocol, no single measurement suffices.^34^ Conventional methods (TEM, DLS, etc.) yield morphology and size but cannot capture dynamic biomolecule binding. High-resolution imaging and spectroscopy can reveal structure and chemistry. Transmission electron microscopy (TEM) and cryo-electron microscopy (cryo-EM) are used to image corona structure. For example, negative-stain TEM of plasma-exposed polystyrene beads shows patchy protein clusters on the surface.^35^ Cryo-EM preserves the native, hydrated state and revealed that plasma proteins form loose clusters both on and between NMs. Such images confirm that some proteins in the “corona” may not be tightly bound to the NM and cautions against overinterpreting bulk proteomics.

Together, these advances illustrate how corona characterization is evolving from static protein inventories to dynamic, system-wide analyses of biomolecular interactions. The next frontier lies in coupling multiomics profiling with high-resolution *in situ* methods and AI-driven analytics to capture coronas as they form, evolve, and direct transformations in real time. Such approaches will not only reveal the mechanistic underpinnings of dissolution, sulfidation, or enzymatic degradation but also generate predictive “fingerprints” that can guide safe-by-design material development. By shifting from descriptive to predictive characterization, the field is poised to transform coronas from experimental artifacts into programmable levers that dictate nanomaterial fate and sustainability.

### 3.2 Computational Modeling and Machine-Learning Predictions

To complement experimental methods, computational tools such as machine learning (ML) are leveraged to predict and elucidate ENM transformations. For example, a recent study used a random forest ML model to predict which proteins from a complex biofluid (yeast) adsorb onto silver NMs, based on the proteins’ physicochemical properties and the NM features.^36^ The model achieved high accuracy (classification AUC ∼ 0.83), and highlighted key variables (protein isoelectric point, NM surface charge, etc.) that govern corona formation. Such data-driven models represent a first step toward predicting NM-specific corona fingerprints and subsequent behaviors without exhaustive wet-lab screening. We are beginning to integrate predictive models, for instance, using known corona profiles to forecast a NM’s cellular uptake or ecotoxicity, aligning with broader efforts in nanoinformatics.^37,38^ Another powerful approach is molecular dynamics (MD) simulations, which offer an atomistic view of corona formation and NM–biomolecule interactions. Recent advances in computing allow simulations of proteins adsorbing to NM surfaces, revealing how proteins orient, unfold, or even competitively exchange (mimicking the experimental “Vroman effect”).^39^ These simulations not only reproduce experimental observations (e.g., confirming that certain proteins bind strongly to specific functionalized surfaces) but also provide molecular insights into how a corona stabilizes a NM in solvent. By comparing simulation outcomes with experimental spectroscopic and microscopic data, we gain confidence in the mechanistic interpretations, bridging the gap between empirical observation and theoretical understanding. Overall, the integration of ML models and MD simulations into corona research enabling predictive safe-by-design strategies, where material formulations can be virtually screened to identify those likely to undergo favorable, benign transformations.

## 4 Corona-Mediated Transformations in Emerging Materials: Case Studies on MOFs

MOFs represent a new frontier for NM applications, and their complex chemistry makes them particularly sensitive to transformation pathways dictated by corona acquisition. MOFs exhibit *structural instability* in complex biological and environmental media: many MOFs readily degrade or transform upon exposure to water, biomolecules, or changes in pH.^5,40^ For example, labile MOFs such as zinc imidazolate frameworks can dissociate in mildly acidic conditions, releasing Zn^2+^ ions *in situ*. This “Trojan horse” effect, ion release upon intracellular dissolution, is a mechanism familiar from metallic NPs and applies equally to MOFs.^41^ In environmental waters, MOF crystallinity can diminish via hydrolysis or linker exchange, leading to partial amorphization or precipitation of metal oxides/carbonates. Crucially, the biomolecular corona that quickly adsorbs onto MOF surfaces influences these transformations. Adsorbed proteins and natural organic matter can either *stabilize* MOFs (by sterically hindering water access to coordination bonds) or *destabilize* them (by chelating metal ions or catalyzing linker hydrolysis). In one study, a Cu-based MOF formed a protein corona rich in fibrinogen when exposed to human plasma, which significantly *diminished its cytotoxicity*,^42^ suggesting that the corona defines the MOF’s biological identity and buffers its reactivity, potentially slowing unwanted rapid disintegration or mitigating acute toxicity. Understanding these unique MOF transformation pathways, from dissolution and ion release to corona-mediated phase changes, is essential as MOFs for drug delivery and environmental remediation are being developed.

The evolving understanding of MOF transformations is deeply informed by lessons learned from traditional engineered nanomaterials (NMs). Many fundamental mechanisms, such as dissolution, aggregation, oxidation, and surface remodeling by biomolecules, have long been studied in metallic and oxide NMs,^3,41,43^ revealing that the protein corona can drastically alter NM fate, by retarding dissolution or catalyzing new phase formation (such as protein-driven sulfidation of silver NMs in serum). Similarly, with MOFs we observe that corona composition (proteins vs metabolites) may direct whether a framework dissolves slowly or transforms into a stable derivative. Another parallel is the importance of particle geometry and coating: just as inorganic NPs can be shielded with coatings to prevent toxic ion leaching, MOFs can be surface modified (e.g., with polymer shells or biomimetic ligands) to moderate their interactions with the surroundings. For instance, coating a Zn-MOF with a thin lipid or polymer layer could delay its contact with water and enzymes. In short, established nanosafety paradigms, such as the Trojan-horse dissolution model and corona-mediated surface chemistry changes could provide a blueprint for anticipating MOF behavior. By applying these insights, researchers can better predict and control MOF transformations, ensuring that this new class of materials is deployed safely and effectively. The current focus is on integrating the responsive characteristics of MOFs with the rich knowledge from colloidal NMs, closing the gap between *innovation* and *safety* in the realm of advanced materials.

Beyond passive biomolecule adsorption, enzyme–MOF interactions drive noteworthy transformation processes with implications for safe-by-design. Certain enzymes can absorb onto MOF surfaces and unfold or become inactivated upon binding, or enzymes may catalyze the breakdown of MOF structures. Our recent study demonstrated that nanoscale MOFs (ZIF-8 and a Cu-imidazolate MOF) induced significant secondary-structure changes in key enzymes such as acetylcholinesterase and α-amylase, reducing the α-helix content and enzymatic activity by over 60% at high MOF doses.^44^ Such perturbation of enzyme structure raises concerns that MOFs could inadvertently inhibit critical biological pathways, highlighting a need for safer designs that minimize nonspecific enzyme adsorption. Conversely, harnessing enzyme interactions can enable biodegradable MOFs, for instance, incorporating peptide-based linkers that specific proteases cleave, thereby triggering MOF disassembly under defined conditions. This strategy allows *programmed dissolution*: a MOF remains stable during use (e.g., circulating in bloodstream or filtering pollutants) but degrades on cue in the presence of a target enzyme or stimulus, releasing its cargo or harmless breakdown products.

Another emerging concept is selective ion trapping by MOFs through corona or linker engineering. Here, MOFs are designed to sequester toxic ions or molecules from their environment and then undergo a controlled transformation to lock those species into stable complexes. For example, a properly functionalized MOF might capture heavy metal ions in its pores and, upon gradual dissolution, precipitate them as insoluble, less bioavailable compounds. Such *engineered transformation pathways* illustrate how we can turn the inherent reactivity of MOFs into an advantage: by programming the acquired corona or structure to achieve benign end-states (e.g., conversion of absorbed ions to nontoxic forms, or timed drug release followed by complete degradation of the carrier). The rich interplay between MOFs and biomolecular coronas highlights the potential for corona-informed material design, which we explore further in the context of safe and sustainable innovation.

## 5 Corona-Informed Design of Safe and Sustainable Nanomaterials

### 5.1 Programmable Transformations through Corona Engineering

Building on our understanding of transformation mechanisms, we explore how biomolecular coronas can be leveraged to design NMs that undergo intentional, safe transformations at the end of their lifecycle. A central theme in translating mechanistic insights into practice is corona engineering—deliberately designing or influencing the biomolecular corona so that it guides NM transformations along safe, desired pathways. Rather than viewing the corona as an uncontrollable byproduct, researchers are developing strategies to *program* it. One approach is molecular imprinting of NM surfaces to create a predisposed corona profile. In this strategy, NM are synthesized or coated in the presence of a “template” biomolecule (for example, a specific plasma protein), forming *cavities or recognition sites* that preferentially rebind that molecule in the future. This has been demonstrated with polymer nanogels imprinted for human serum albumin (HSA): upon introduction into blood, the nanogels selectively cloaked themselves with HSA, yielding a benign, “stealth” corona dominated by albumin.^45^ By favoring a single abundant protein, the imprinting approach can reduce opsonization by immune proteins and modulate downstream transformations since HSA is known to protect NMs from agglomeration and oxidative attack. More broadly, one can imagine tuning the corona to include proteins that actively drive a beneficial transformation.

We propose “*corona-informed NM design*”, where if a particular protein is known to detoxify a NM (for instance, by catalyzing a surface reaction like sulfidation or by sequestering released ions), the NM’s surface could be functionalized to preferentially attract that protein. Conversely, if a certain surface composition tends to recruit proteins that promote an undesirable reaction (e.g., proteins that accelerate dissolution into toxic ions), a safe-by-design approach would avoid coating or include a blocking ligand to steer the corona composition away from that outcome. This level of control extends beyond proteins: small-molecule coronas (metabolites, cofactors) might be engineered via precoating NMs with specific ligands or surfactants, effectively imprinting a layer of molecules that can later exchange with environmental ones in a predictable fashion. The ultimate goal is a programmable transformation: materials that, by virtue of their tailored corona, age gracefully—for example, by breaking down after a set period or by neutralizing their byproducts via corona-catalyzed reactions. Together, these principles can be synthesized into a roadmap for corona-informed design, highlighting how mechanistic insights into biomolecular coronas translate into predictive tools, safe-by-design strategies, and policy alignment (Figure 5).

**Figure 5:**
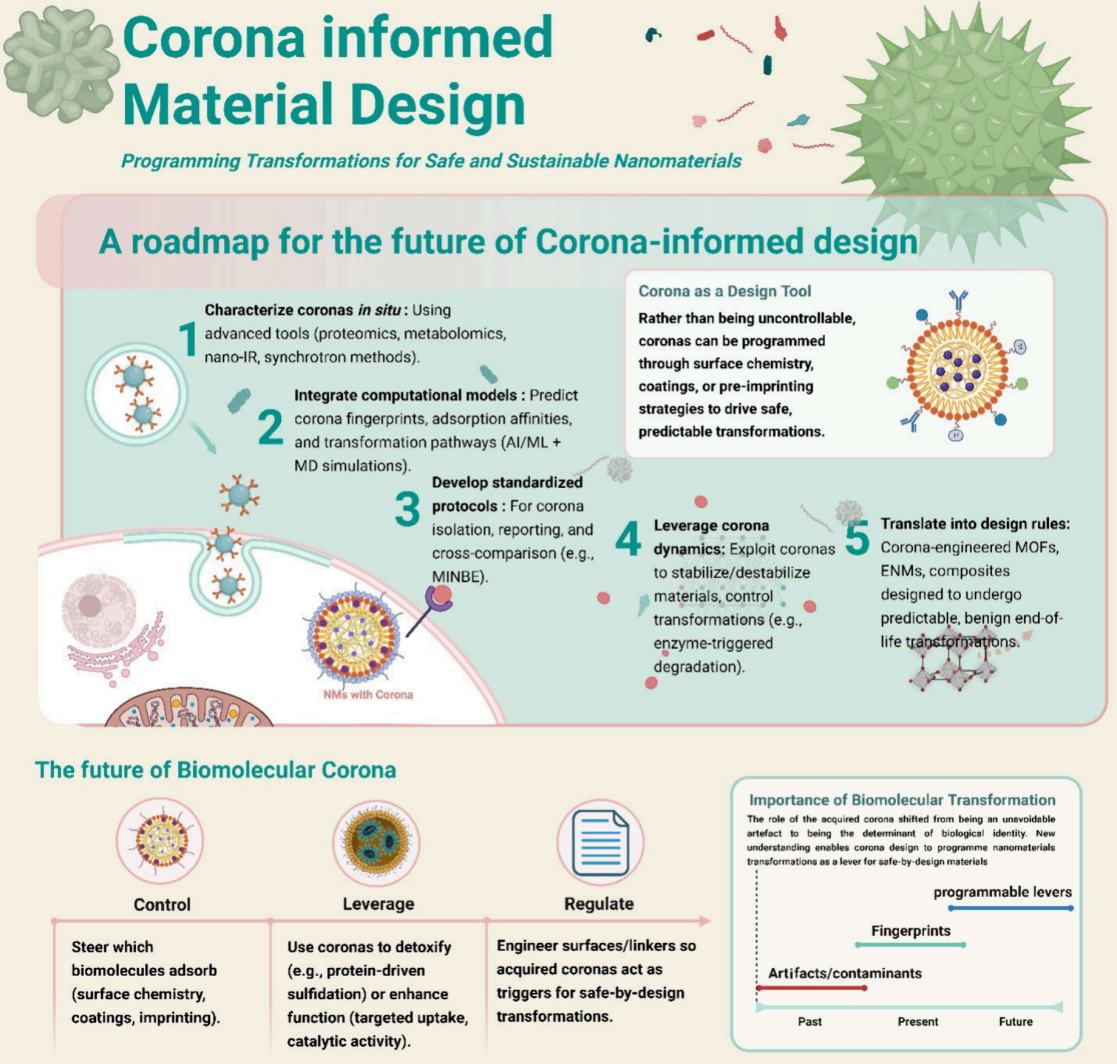
Corona-informed materials design roadmap. Embedding biomolecular corona considerations into (nano)­material development enables safe and sustainable design. The roadmap outlines five steps: *in situ* characterization, predictive modeling, standardization, leveraging corona dynamics, and translation into design rules. Future directions include controlling biomolecule adsorption, exploiting coronas to stabilize/detoxify materials, and programming material surfaces so that acquired and evolved coronas trigger safe transformations at the desired time/location. The timeline highlights the evolving role of coronas, from unavoidable artifacts to determinants of biological identity to programmable levers for safe-by-design nanomaterials that undergo corona-controlled transformations on demand. Figure created using Biorender Software.

### 5.2 Case Studies in Safe-by-Design: From Biomedicine to Environment

Implementing these design principles requires case-by-case tailoring, and recent examples illustrate how safe-by-design NMs are taking shape. In the biomedical realm, MOF-based drug delivery systems have embraced the idea of programmed transformations. One example utilized an enzyme-responsive MOF for cancer therapy: a Zr-based MOF (UiO-66 type) was modified with peptide linkers that remain intact in circulation but are cleaved by a tumor-associated enzyme, causing the MOF to disintegrate and release its drug payload specifically at the tumor site.^46^ Here the biomolecular trigger (an enzyme overexpressed in the tumor microenvironment) effectively dictated the transformation of the carrier into inactive fragments at the right time and place. Another example is the design of biodegradable MOFs for imaging, using nutritionally benign^47^ metals like iron or magnesium,^47^ which function as contrast agents or vectors and then dissolve into nontoxic species (e.g., Fe^3+^ ions integrate into the body’s iron stores) over days, reducing long-term accumulation. The biomolecular corona in such systems is often engineered by coating the MOF with a lipid or peptide that both improves biocompatibility and controls the degradation rate (e.g., a peptide that slowly hydrolyzes to trigger MOF dissolution *in vivo*). In environmental applications, a compelling safe-by-design case is photocatalytic MOF composites for water remediation^48^ that degrade organic pollutants and then self-degrade under sunlight, leaving behind only mineralized end-products. The corona can assist here too: by absorbing NOM from the water, the MOF’s surface reactivity can be modulated such that once pollutants are removed, the NOM corona induces a phase change (e.g., converting the MOF to an oxide that sediments out). Another example involved a NM for agriculture: a nanoscale silica carrier was functionalized to bind a specific soil enzyme; after delivering micronutrients to plants,^49^ the enzyme in soil gradually digested the carrier’s biopolymer coating, causing the NM to disassemble into soluble, innocuous components. The common thread is that the materials were designed with their end-of-life in mind. By integrating triggers (enzymatic, chemical, or photonic) into the material’s lifecycle, and capitalizing on the inevitable biomolecular interactions, NMs can be designed to transition into harmless forms after serving their purpose. MOFs, with their inherently modular chemistry, are ideal model systems to demonstrate these concepts, since swapping linkers or nodes adjusts degradability and coronas can fine-tune interactions. Such case studies provide real-world validation that sustainability and performance can be co-optimized, which feeds back into the design loop.

### 5.3 Green Nanotechnology: Industry and Policy Outlook

As the science of biomolecule-driven transformations matures, its translation into industry and policy is accelerating. The SSbD framework, embedding environmental safety at the earliest stages of material innovation, is championed by regulatory bodies and funders worldwide. The European Commission and the OECD have outlined roadmaps requiring developers to assess and mitigate risks before commercialization rather than retroactively. Corona-induced transformations provide essential insights for these assessments: if a nano-MOF acquires an eco-corona in wastewater that prolongs persistence, its formulation can be redesigned or coated with benign stabilizers to alter that trajectory.

Green nanotechnology builds on this ethos by selecting biodegradable linkers and earth-abundant, nontoxic metal centers, reducing risks if MOFs escape into ecosystems. Such strategies appeal to industry because they align with circular economy principles and reduce liabilities. Materials engineered to self-dismantle into harmless constituents removes the need for costly remediation at end-of-life. Lifecycle assessments (LCA) increasingly quantify these benefits, often showing that degradable materials outperform persistent ones. For instance, a photocatalytic MOF that degrades into sand-like residues may score far higher in LCA metrics than an inert catalyst that accumulates and demands energy-intensive retrieval. Policy is moving toward certification of nanoenabled products that meet safety and sustainability benchmarks. In the near future, nanomedicines or agrochemicals may carry labels verifying decomposition into nontoxic residues. Achieving such credibility depends on robust science, much of it rooted in understanding biomolecular coronas and their role in directing materials transformations.

We argue that coronas should be viewed as programmable levers for guiding (nano)­material fate, activity, and safety. We hope this Account inspires the broader community to embrace biomolecular coronas and biomolecular transformations as a foundational design principle, the next generation of nanomaterials can be effective, intrinsically safe, adaptable, and sustainable.
